# Promoting Good Public Governance and Environmental Support for Sustainable Economic Development

**DOI:** 10.3390/ijerph16244940

**Published:** 2019-12-06

**Authors:** Gratiela Georgiana Noja, Mirela Cristea, Nicoleta Sirghi, Camelia-Daniela Hategan, Paolo D’Anselmi

**Affiliations:** 1 East European Center for Research in Economics and Business, Department of Marketing and International Economic Relations, Faculty of Economics and Business Administration, West University of Timisoara, 16 Pestalozzi Street, 300115 Timisoara, Romania; gratiela.noja@e-uvt.ro; 2 Department of Finance, Banking and Economic Analysis, Faculty of Economics and Business Administration, University of Craiova, 13 A I Cuza Street, 200585 Craiova, Romania; 3 Department of Economics and Economic Modelling, Faculty of Economics and Business Administration, West University of Timisoara, 16 Pestalozzi Street, 300115 Timisoara, Romania; nicoleta.sirghi@e-uvt.ro; 4 Department of Accounting and Audit, Faculty of Economics and Business Administration, West University of Timisoara, 16 Pestalozzi Street, 300115 Timisoara, Romania; camelia.hategan@e-uvt.ro; 5 Guildhall School of Business and Law, London Metropolitan University, 66-220 Holloway Rd, London N7 8DB, UK; paolodanselmi@gmail.com

**Keywords:** good governance, public administration, environmental support, economic development, poverty

## Abstract

Good governance promotes the fundamental grounds of participation and democracy in contemporary public administration, whilst institution building and the (in)effectiveness of public administration is linked to economic growth. This synergy brings forth sheer implications on the sustainable economic development. On this composite setting, the paper examines several fundamental credentials of public administration in the European Union (EU) countries, in relation to economic development, but also poverty, research, and development support, as representative socio-economic credentials. The empirical analysis is based on data covering the 1995–2017 lapse of time, processed through three econometric procedures, namely robust regression, structural equation modeling, and Gaussian graphical models. The main results emphasize that there are significant joint implications of public administration on the considered socio-economic dimensions. General government spending and, particularly, the environmental support, have positive implications on the European Union economies, leading to significant increases in the Gross Domestic Product (GDP) per capita and downsize in poverty risk (more emphasized in regard to the government expenditures than the environmental protection). Overall, the quality of governance in EU countries requires an additional effort dedicated to leverage good public governance in order to support the long-term economic development.

## 1. Introduction

The concept of good governance, as developed by the World Bank, is a reference point on how a country’s dominant administrative structure can be measured. Good governance has eight major characteristics, respectively “participation, respect for the rule of law, transparency, responsiveness, consensus-oriented, equity and inclusiveness, effectiveness and efficiency, accountability” [1] (p. 1). In order to be effective and sustainable, good governance must be anchored in a vigorous democracy that respects the rule of law, freedom of the press, respect for society’s organizations, as well as efficient and independent public bodies [2].

As evolution landmarks, the concept of good governance was representative for developed countries around the world. Since developing countries was confronted in the 1980s with debt crises [3] and continued in the mid-1990s, the concept of good governance has evolved and has become a guide for developing countries as well. Moreover, government reforms in beneficiary developing countries have become a prerequisite for the financial assistance from international donor institutions [4], and good governance has been enforced as a requirement for national states with the role of carrying out economic and administrative reforms [5].

Altogether, the development of the economies also brings with it worldwide environmental problems that are reaching higher significance, due to the gravity of the effects that they produce throughout the entire society. Thereby, environmental governance depicts a substantial element of the national governance organization [6,7]. In 1987, Brundtland famously defined sustainable development as “the development that meets the needs of the present without comprising the ability of future generations to meet their own needs” and popularized the term “sustainable development” [8] (p. 41). The link between government environmental support and sustainable development is testified by the United Nations Sustainable Development Goals (SDGs) [9], which define a paradigm for sustainable development.

Consequently, there is an affluence of studies that have deepened the good governance dimensions in relation to economic development [10,11,12,13,14,15,16], using different methodology, but none of them have focused on the environmental protection impact at the same time. 

In this frame of reference, the objective of our paper is to assess the implications of public administration, with a particular focus on the environmental protection component and public governance credentials (the well-known Worldwide Governance Indicators, WGI), upon economic and social development, within the European Union (EU) countries, for the 1995–2017 lapse of time. We aim to explore what is meant by "good public governance" and "sustainable development" and to examine some fundamental beliefs of the public administration in the EU Member States (MS), regarding the sustainable economic development.

For the research endeavor, we have built up several econometric procedures: the macro-econometric models—robust regression (RREG) technique and simple and multifactorial models, in order to assess the direct implications of public administration credentials upon each considered dependent variables. Structural equation modeling (SEM) and Gaussian graphical models (GGM), to account for the interdependencies between all considered variables. We appraise five specific research hypotheses in order to check for the direct implications of public administration dimensions upon economic development, poverty, and research and development (R and D) support, both single, and joint implications, as well as the overall (direct, indirect, total) interlinkages of public administration credentials upon economic and social development (upturn in welfare state and downsize of poverty). Public support for environmental protection holds a distinct evaluation within these research hypotheses.

The paper enriches the existing literature with an updated and integrative analysis of the good public governance perceptions, on the one hand, and general and environmental support on the other hand, for sustainable economic development of EU countries. 

After a brief introduction appraising the general context and significance of this topical subject, the remainder of the paper is settled as follows. Section 2 tackles the main findings and diverse strands of thought in the literature, where we outline the most significant studies on the concept of good governance and public administration effects on economic development. In Section 3, we draw up the data and methodology used for the econometric analysis, with a keen center on macro-econometric models, structural equation, and Gaussian graphical models. Section 4 discloses the results obtained by means of each econometric procedure and carries forth an inclusive discussion of their impacts. Section 5 comprises the conclusive remarks, followed by supplementary information that support the empirical research, retrieved in the Appendix A. 

## 2. Literature Review 

The public administration, with a focus on good governance, and its link with economic and social development, has received a great deal of attention in the scientific literature, both as regards the consent of its meaning, and regional or national effects.

### 2.1. The Concept of Governance and the Public Administration 

Scholars of public law and political theory have been revising Montesquieu’s model of the separation of powers nearly three centuries after its original formulation in 1748. Likewise, public law appears to be based on the Weberian ideal typical view of rational behavior of public administration, a view that has also been revised over the course of the last century by both management sciences and micro-economics. The Max Weber [17] model is the basis for the neoclassical model of the profit-maximizing firm, and it is also the basis for constitutional and administrative law and public administration organization. Katsamunska [18] considered a different approach of the concept of governance within the field of public administration theory, starting from the Weberian public administration model to the good governance concept, as developed by the World Bank and International Monetary Fund (IMF).

By analyzing the groundings of the concept of governance in the field of public administration, we have noted several approaches. Some show that public administration tends to outline anti-bureaucratic and anti-government sentiments, preferences for limited governance [19]. Other models express state-centered and jurisdictional understandings of governance, in which public administration is dependent on rules and laws [20].

The general notion about the concept of governance within the field of public administration theory is that “for managing democratic government, there is the interrupted triangle of governance. The citizen-customer-voter-taxpayer (bottom left corner of the triangle: citizens) does not enter into a direct relationship with the government employee (bottom right corner of the triangle: public administration), whom he pays through his taxes. Within a democracy, politicians (top corner of the triangle) mediate this relationship and, in turn, oversee public administration towards the ends and objectives expressed by the same citizens” [21] (p. 104). Between the citizens and the politicians and between the politicians and the public administration, “some sort of negotiation” takes place “which, if the transitive property is applied, should guarantee a dialectic relationship between public administration and the citizen, albeit mediated by the politician. It is necessary to understand whether this model corresponds to reality” [21] (p. 104), i.e., if the conduct of public administration under the aegis of politics is going to yield government effectiveness, rule of law, and control of corruption [22].

While the governance is unanimously accepted, representing “the process of decision-making and the process by which decisions are implemented (or not implemented)” [1], as regards the concept of good governance, Keefer [23] (p. 439) considered that there is "no agreed definition of governance that would provide a convenient device for organizing the literature". Gisselquist [24] summarized the definitions of good governance from the perspective of donor institutions, respectively United Nations (UN), multilateral development banks, European Commission, IMF, and the Organization for Economic Co-operation and Development (OECD). They found that the definitions differ from one institution to another in terms of measuring the quality of governance. Diarra and Plane [25] studied the issue of governance within the World Bank and its impact on the good governance pattern, and suggested a shift from focusing basically on economic issues of governance towards progressively emphasizing the political dimensions.

The approach of good governance involves the identification of an optimal system of governance, which, through its efficiency, leads to long-term economic prosperity [26]. In addition, good governance represents active and effective cooperation between state authorities and citizens, as participants in the process of public administration. As attested by Kaufmann et al. [27] (p. 2), public governance is measured for each of its six wide dimensions, known as the WGI, namely: “Control of corruption, political stability and absence of violence, government effectiveness, regulatory quality, rule of law, and voice and accountability”. The WGIs have been determined since 1996 by the World Bank, in two ways, namely: “(1) in their standard normal units, ranging from approximately −2.5 to 2.5, and (2) in percentile rank terms from 0 to 100, with higher values corresponding to better governance” [27] (p. 2). By means of WGIs, there are possible relevant and basic comparisons among around the world as regards the perception of good governance by citizens of each of the 215 countries of the world, for which the WGIs are determined [28].

### 2.2. Public Governance and Sustainable Development

The quality of governance is influenced by a number of factors, one of which has recently been relevant to the economic crisis. The results of AlBassam’s [29] research, during the economic crisis, revealed a fluctuating interlinkage between governance and economic growth, which leads to the requirement for long-term strategies to advance good governance application. 

As regards the role of good governance in economic growth, there have been several opinions for the developed countries [10], on the one hand, and in the struggle for poverty mitigation, and upright economic growth for developing countries [12] on the other hand. Grindle [30] showed that the individual elements of good governance can be benchmarks in the process of modernizing governments, but they are also influenced by the own environment of each country, political, economic, or cultural.

Most of the studies, focused on the relationship between public governance and economic growth, have considered the WGIs for public governance measure, and have generally concluded that the components of public governance have a positive impact on economic growth [13,14,15]. Previous studies show that in EU countries, there are divergences in respect of environmental regulations, which are mainly based on geographical differences [31]. Ferreiro et al. [32] conducted a study on environmental protection spending and found that almost half of EU countries invest in environmental protection, but did not find a standard fiscal policy model that would stimulate economic growth and competitiveness, and at the same time, to contribute to the well-being of the population [33]. On the other hand, Apergis et al. [34] show that public expenditures on the environment in European countries are not convergent, even though the EU has issued several important regulations regarding the protection of the environment, which indicates that it is necessary for public spending policies to be better coordinated for a sustainable development.

Bayar [11] examined the impact of the WGIs on economic growth in the EU’s transition economies, for the 2002–2013 timespan. The results showed that all governance indicators, except for the quality of the regulations, had a statistically significant and positive impact on the economic growth. The data also indicated that corruption control and the rule of law had the greatest impact on economic growth, while political stability had the smallest influence. Graf Lambsdorff [35], based on the corruption perceptions index (CPI), considered that the public sector is grasped not only by corruption, but also by the quality of governance.

Merloni [36] (p. 107), cross-examining the public administration in European countries with similar dimensions as regards the size of the population and level of economic development, with a specific focus on Italy, concluded that “the basic factor driving this complexity is the country’s size of population relative to its level of social and economic development”. At the same time, Ardielli [37] appraised the good governance of the EU countries and pointed out countries that were flourishing in the long term, especially the Nordic states—Finland, Sweden, and Denmark, as well as countries with higher deficiencies in terms of good governance, such as Romania, Bulgaria, or Greece.

Marino et al. [38] explored the connection between the WGIs and the socio-economic development indicators of the BRICS countries (namely, Brazil, Russia, India, China, and South Africa). The results suggested a positive relationship between the human development index (HDI) and the indicators on the efficiency and control of government corruption, on the one hand. On the other hand, the implications of the WGIs upon gross domestic product (GDP) revealed a positive impact as regards the corruption control indicator, and a negative one as for voice and responsibility, and the other indicators of political stability.

Grindle [12] shows that the good governance has become a paramount strategy for reducing poverty, following the structural disappearance of the adjustment policies, promoted by the IMF and the World Bank in the 1980s and 1990s. Opposite, Kwon and Kim [39] (p. 353) established a causal link between good governance and poverty reduction, out of the six WGIs tested, and “the empirical evidence does not support the hypothesis that good governance leads to poverty reduction”. Gough et al. [40] show that the absence of effective state institutions can create a major obstacle to the development of a country, which is why good governance of these institutions is primarily needed for socio-economic development. On these lines, in 2015, the UN [9], through the 2030 Agenda for Sustainable Development to achieve the 17 goals, primarily targeted poverty eradication, reaching the needs of countries within the ecological boundaries of the planet. 

Chen and Ganapin [41] highlight the regional intergovernmental approach and advocate for a polycentric governance approach. As regards the allocation of the environmental expenditures at the level of the EU, there isn’t “a homogeneous model”, due to “specific circumstances and priorities” [42] (p. 1145) of each country. Delgado-Serrano et al. [43] show that social-ecological systems around the world are supported by natural resources belonging to the entire community. As regards the interlinkages between good governance, the environmental expenditures and economic growth, Gil et al. [44] (p. 1111) proved that, in Asian developing countries, almost all the WGI’s components act as incentives of economic development, “by strengthening the rule of law, improving quality of policy formulation, policy implementation, and quality of contract enforcement”. Zhang and Chen [45] (p. 19) investigated the impact of public participation on polluting emissions and concluded that “environmental governance requires participation from multiple subjects”.

From an organizational point of view, all authors imply a synergy between government effectiveness and government expenditure, thus implicitly adopting a Weberian hypothesis (WH) about public administration’s organizational behavior, i.e., public administration as a bureaucracy is rational and highly efficient [46].

Consequently, summing-up the relevant findings in the literature, we emphasize that: the WGIs are the most used variables to measure good governance, most of the studies pointed out the growing effects of good governance upon economic development, each component of the WGI acts differently on economic growth, there are opposite results as regards the influence of good public governance upon poverty, and public environmental protection support was less considered for assessing its impact upon economic growth, although it was included as a distinct goal for sustainable development.

## 3. Materials and Methods 

In order to measure the public administration implications upon economic and social development, based on the literature review, we have selected several indicators that disclose, on the one hand, the share of general government expenditure on GDP, focusing on the environmental protection component, as well as the credentials of the public administration activities, namely public governance, on the other hand. As regards the economic and social development, we account for three implications: enhancing GDP per capita (for economic development), reducing poverty (for the main social impact), and heightening R and D expenditures (for technological and innovation support).

The contribution of government expenses to GDP follows the “Classification of the functions of government (COFOG)”, which was enhanced by the OECD, being revised under the current form in 2019 [47]. Accordingly, the general government expenditure comprises ten main classes (known as “COFOG I level”), namely “general public services, defense, public order and safety, economic affairs, environmental protection, housing and community affairs, health, recreation, culture, and religion, education, and social protection” [48] (p. 37). Furthermore, as regards the component of environmental protection expenses, COFOG includes the following expenditures: “waste management, wastewater management, pollution abatement, protection of biodiversity and landscape, R and D environmental protection, and environmental protection not elsewhere classified (n.e.c.)” [48] (p. 37).

In order to weigh public governance, namely the practices and activities of public authorities conducted by their institutions, we used the WGIs generated in the standard normal units (ranks between −2.5–2.5). The meaning of each WGI dimension is synthesized in Table 1 and summary statistics of all indicators are described in Table A1 (in the Appendix A). 

Similar indicators and methodological credentials were also used by other authors focused on this topical subject, such as Bayar [11], Marino et al. [38], Kwon and Kim [39], and Gil et al. [44].

Consequently, the dataset compiled for our research covers all EU-28 MS and the 1995–2017 lapse of time and grasps representative indicators for the EU countries on public administration field and other socio-economic indicators, with a specific focus on environmental protection and economic development, as follows (Table 1):

- Public administration indicators: Total general government expenditure (*Gen_GOV_exp*), expenditure of general government on environmental protection (*Env_GOV_exp*), control of corruption (*COR_CTRL*), government effectiveness (*GOV_effect*), political stability and absence of violence/terrorism (*POL_stab*), regulatory quality (*REG_quality*), rule of law (*Rule_law*), and voice and accountability (*Voice_acc*).
- Socio-economic indicators: Total GDP (*GDP_tot*), GDP per capita (*GDP_cap*), people at risk of poverty or social exclusion (*POV*), research and development (*R and D*) expenditures (*GERD*), tertiary education 30–34 years (*EDU_Tert),* educational attainment for upper secondary and post-secondary non-tertiary education *(Edu_att*), annual net earnings (*Net_earn),* and employment rate *(ER)*.

The data were extracted from Eurostat [49], OECD databases [50], and The Worldwide Governance Indicators (WGI) [28].

**Figure 1:**
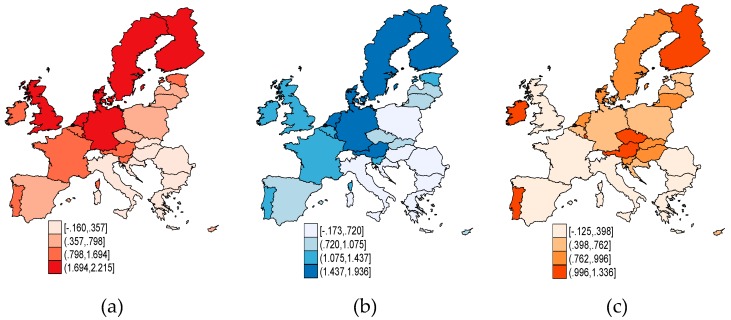
Public governance credentials in European Union (EU) countries, 2017: (**a**) *COR_CTRL*, (**b**) *GOV_effect*, and (**c**) *POL_stab*. Source: Own contribution in Stata.

**Figure 2:**
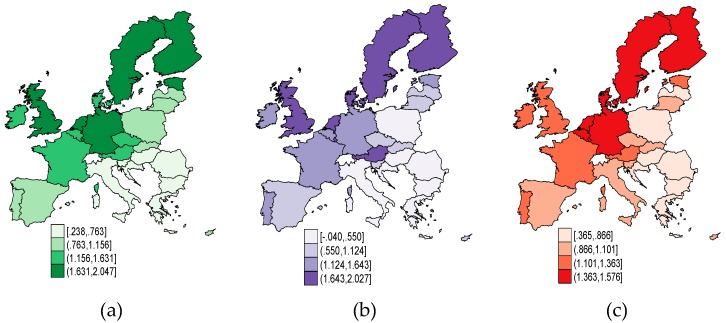
Public governance credentials in EU countries, 2017: (**a**) *REG_quality*, (**b**) *Rule_law*, and (**c**) *Voice_acc*. Source: Own contribution in Stata.

In Figure 1 and Figure 2, we have mapped each dimension of public governance across the EU, expressed by WGI, in 2017. As regards corruption control (Figure 1a) and government effectiveness (Figure 1b), the strapping outcomes are achieved by the Nordic Sates (Denmark, Finland, Sweden), along with Germany, the United Kingdom, the Netherlands, and Austria. Together with Estonia, these countries also entailed a greater perception as regards the regulatory quality (Figure 2a), but also a high degree of confidence in the rules of society (Figure 2b) and voice and accountability (Figure 2c). To the contrary, the EU countries in Central and Eastern Europe (CEE), but also Italy and Spain, counted the lowest-ranking outcomes of public governance. The only dimension of WGI which is steadier in CEE countries (Hungary, Czech Republic, Slovenia, Slovak Republic, and Lithuania) than in other EU countries (like France and the UK), is the political stability (Figure 1c). The highest political stability is attained in Finland, Austria, Ireland, and Portugal.

In order to provide appropriate correspondence among variables, the selected indicators have been stationary through logarithm (unit-root test results are presented in the Appendix A, Table A2). Further, we have applied the research methodology that covers three econometric procedures, namely: (1) macro-econometric models—robust regression (RREG) technique, in order to assess direct implications of public administration credentials upon each dependent variable, GDP per capita, poverty, and R and D support (unifactorial models), as well as the joint direct influence on several socio-economic credentials (multifactorial models), (2) structural equation modeling (SEM), in order to analyze the direct, indirect, and total interlinkages between the public administration credentials, GDP per capita, and poverty, and (3) Gaussian graphical models (GGM), to account for the interdependencies between all considered variables.

Firstly, we have configured three sets of eight simple regression models that were processed through the robust regression (RREG) technique and eight other multiple regression models (robust) that account for the joint direct influence of public administration credentials on several socio-economic dimensions. Robust regression (RREG) detaches the outliers or high leverage data points in the sample (hence the influential points are dropped), based on Cook’s distance (Cook’s D) and two types of weights on the iteration process (Huber and Biweight weighting).

**Figure 3:**
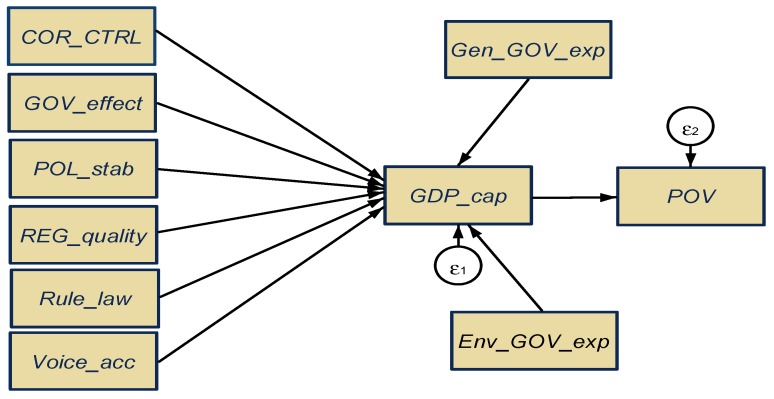
General configuration of the structural equations modeling (SEM) model. Source: Own contribution in Stata. Note: *COR_CTRL*—control of corruption; *GOV_effect*—government effectiveness; *POL_stab*—political stability and absence of violence/terrorism; *REG_quality*—regulatory quality; *Rule_law*—rule of law; *Voice_acc*—voice and accountability; *Gen_GOV_exp*—total general government expenditures; *Env_GOV_exp*—expenditure of general government on environmental protection; *GDP_cap*—Gross Domestic Product per capita; *POV*—people at risk of poverty or social exclusion.

Second, we have assessed the overall interlinkages (direct, indirect, and total) from a dual presumption (the determinants-impact interplay), by applying the structural equations modeling (SEM) procedure, for estimating the public administration impacts upon GDP per capita and poverty, as it is shown in Figure 3.

Following our general objective, we check for the following hypotheses (H):

- H_1_: There are significant direct implications of public administration dimensions upon economic development.
- H_2_: There are significant direct implications of public administration dimensions upon poverty.
- H_3_: There are significant direct implications of public administration dimensions for R and D support.
- H_4_: There are direct joint implications of public administration dimensions on socio-economic credentials (economic development/welfare, education and development, labor market outcomes).
- H_5_: There are overall (direct, indirect, total) significant implications/interlinkages of public administration credentials upon economic and social development (upturn in welfare state and downsize of poverty).

## 4. Results and discussion

### 4.1. Macro-Econometric Models

In order to verify our first hypothesis and to assess to what extent the public administration credentials influence the economic development (proxied by the GDP per capita, as the dependent variable), at the EU level, we have built up a macro-econometric model, processed through RREG, for each dimension of public governance (eight models in total), as independent variables (Table 2). 

The results obtained for assessing the implications of each of the eight public governance variables, which grasp the dimensions of public administration, show that the most significant influence on GDP per capita (74.9%) is explained by government effectiveness (*GOV_effect*) (a coefficient of determination, *R*^2^, of 0.749), for a total number of 507 observations (*N*). Strong linkages are also entailed between the GDP per capita and the soundness of the rule of law (*Rule_law*, 72.1%), enhanced corruption control (*COR_CTRL*, 67.3%), and stronger public voice (*Voice_acc*, 63.8%), but their estimated impact is of a lower intensity.

We note that all the dimensions of public administration within the EU (models 1 to 8) have a favorable influence on economic development (highly statistically significant at the threshold of 0.1% and 1%), being in line with the results of Bayar [11] (for the EU’s transition economies) and opposite to Marino et al. [38] (for the BRICS countries). The strongest positive impact on GDP per capita is exercised by the total general government expenditure (*Gen_GOV_exp*) (the estimated coefficient is positive, 2.347, and extremely statistically significant at the 0.1% threshold). As regards public environmental protection expenses (*Env_GOV_exp*), the influence on GDP per capita is significant (the estimated coefficient is positive, 0.144, and statistically significant at the 1% threshold), although lower than in the case of the other variables. This result attracts the recommendation, mainly, for public authorities to strengthen the support granted for environmental protection [45]. Out of the six dimensions of public governance, the perceptions of people in deciding on their government, and the freedom of expression, association, and media (*Voice_acc*) showed the highest direct effect on the degree of economic development (*GDP_cap*), but also the perceptions of the government’s ability to enforce reliable policies and regulations in order to sustain private sector development (*REG_quality*). Special attention among the six dimensions has to be given to political stability and/or politically motivated violence, including terrorism (*POL_stab*) (since the estimated coefficient is the lowest among all of them, 0.416, extremely statistically significant at the 0.1% threshold), as also proven by Bayar [11].

Thus, we can say that, the first hypothesis, H1: There are significant direct implications of public administration dimensions upon economic development, is fulfilled, being registered favorable impacts upon GDP per capita for all considered variables.

In order to assess our second hypothesis, respectively, in what extent the public administration credentials affect poverty ratios, at the EU level, we have processed another set of eight macro-econometric models for each dimension of public administration (independent variables, model 1 to 8), in direct relation with poverty (as the dependent variable) (Table 3). 

The results (Table 3) reveal that the most significant share in influencing poverty (59.2%) is explained by the perceptions of people in deciding on their government, and the freedom of expression, association, and media (*Voice_acc*) (a coefficient of determination, *R*^2^, of 0.592), from a total number of 378 variables (*N*). 

A number of seven variables of public administration (models 1 to 7) have registered favorable influences in terms of poverty reduction (negative coefficients and highly statistically significant at the threshold of 0.1%), as Grindle [12] also proved, but contrary to Kwon and Kim [39]. The most significant impact on poverty’s reduction, at the level of EU countries, is also induced by the total general government expenditure (*Gen_GOV_exp*) (the estimated coefficient is negative, –0.772, and extremely statistically significant at the 0.1% threshold), as in the case of previous macro-econometric models (H_1_). At the same time, however, the implications of the government contribution for environmental protection (*Env_GOV_exp*) is not significant for poverty reduction (the estimated coefficient is positive and not statistically significant). This involves a higher implication of government authorities in supporting environmental protection, as the results of H_1_ also revealed. 

Among the six dimensions of public governance, the perceptions of people in deciding on their government, and the freedom of expression, association, and media (*Voice_acc*) induced the most substantial reduction of poverty (model 6), but also the perceptions of the government’s ability to enforce reliable policies and regulations in order to sustain private sector development (*REG_quality*). These results are similar to those obtained in the case of economic development implications (H_1_), which reconfirm that they are significant for the EU member states’ economic and social development. A targeted assistance among the six dimensions of public administration is recommended for the perceptions of public authorities’ implications for private benefit, including different types of corruption (*COR_CTRL*) (since the estimated coefficient is the lowest among all of them, –0.167, extremely statistically significant at the 0.1% threshold).

Thus, we can attest that, the second hypothesis, H_2_: There are significant direct implications of public administration dimensions upon poverty, is partially fulfilled (except for the government support for environmental protection).

As regards our third hypothesis, respectively, H_3_: in what extent the public administration credentials stand by the R and D contributions to technological changes, at the EU level, we have rebuilt the macro-econometric model to account for R and D expenses (as the dependent variable), in relation with each dimension of public administration (independent variables, model 1 to 8) (Table 4). 

In this particular case (Table 4), all public administration dimensions have registered favorable impacts upon R and D support, more visible for the total general government expenditure effects (*Gen_GOV_exp*) (the estimated coefficient is positive, 2.679, and extremely statistically significant at the 0.1% threshold). Similar to results obtained for the H_1_ and H_2_ hypotheses, the government contribution for environmental protection (*Env_GOV_exp*) does not have a keen impact on R and D contribution (the estimated coefficient is positive, yet without significance from a statistical point of view). Among the six dimensions of WGI, the perceptions of people in deciding on their government, and the freedom of expression, association, and media (*Voice_acc*) directly influence the enhancement of R and D contribution of GDP (model 6), and the lowest (but favorable and statistically significant) for the political stability perceptions (*POL_stab*) (model 3). Thereby, we can attest that, the third hypothesis, H_3_: There are significant direct implications of public administration dimensions on R and D support, is fulfilled.

We went further with the research endeavor to configure a new set of multifactorial econometric methods which enhance the joint influence of all public governance credentials on the economic activity and welfare/poverty (proxied by *GDP_tot*, *GDP_cap*, *POV*), upon the educational and R and D credentials (proxied through *GERD*, *Edu_att*, *Edu_tert*), as well as on the labor market outcomes (proxied by *Empl_rate* and *Net_earn*). The results are synthesized in Table 5.

We note that an increased joint impact of public governance credentials is induced on all selected socio-economic dimensions, particularly on the GDP per capita, as well as on poverty levels, research and development support, and labor market outcomes, as entailed by higher values of the R-squared. Hence, in the case of welfare impacts of public administration, empirical results show that 81% of the variation in GDP per capita in EU countries can be explained by the variation in public governance credentials. In this case, positive effects are generated especially by the government effectiveness (the estimated coefficient is 0.958, extremely significant at the 0.1% threshold), as well as by an increased corruption control (coefficient of 0.05), higher general (coefficient of 0.62), and environmental (coefficient of 0.04) government expenditures. In almost all considered cases, government effectiveness has significant positive implications, along with corruption control, and regulatory quality (notably in relation with R and D, education, and labor market outcomes).

Thus, we can attest that H_4_: There are direct joint implications of public administration dimensions on socio-economic credentials (economic development/welfare, education and development, labor market outcomes), is fulfilled.

Overall, each public administration dimension represents a significant keystone for further economic development enhancing (H_1_), poverty downsizing (H_2_), or R and D support (H_3_). As regards the joint implications of public administration dimensions upon socio-economic credentials (H_4_), the most prominent are for government expenditure and government effectiveness in relation to economic development, poverty, and R and D assistance. 

### 4.2. Structural Equations Modeling (SEM) results

**Figure 4:**
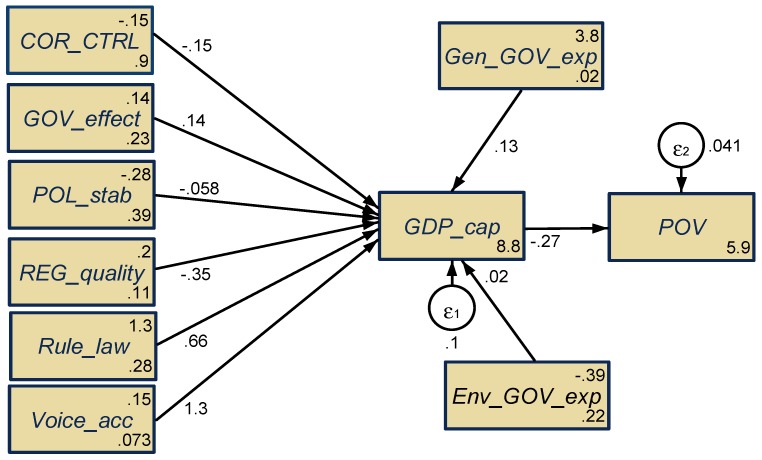
Results of the SEM model for the EU-28 countries, 1995–2017. Source: Own contribution in Stata.

As regards the fourth hypothesis, respectively, H_4_: to what extent do all considered public administration variables, jointly influence (direct, indirect, total) the economic development and poverty levels, we have configured the SEM model, as shown in Figure 4, estimated by the maximum likelihood procedure (results of several tests applied for the validity and scale reliability are presented in the Appendix A, Table A3, Table A4 and Table A5).

The integrative results gathered through SEM as regards the effects of public administration dimensions on economic development, revealed unfavorable impacts in the case of the following public governance dimensions: that the perceptions of public authorities’ implications for private benefit, including different types of corruption (*COR_CTRL*) (the estimated coefficient is negative, –0.15, extremely statistically significant at the 0.1% threshold), being in line with the results obtained by Bayar [11]. The perceptions of the government’s ability to enforce reliable policies and regulations in order to sustain private sector development (*REG_quality*) (the estimated coefficient is negative, –0.35, highly statistically significant at the 1% threshold), and the political stability perceptions (*POL_stab*) (the estimated coefficient is negative, –0.058, although not statistically significant). Favorable implications upon GDP per capita were induced by the following WGI dimensions: perceptions of people in deciding on their government, and the freedom of expression, association, and media (*Voice_acc*) (the estimated coefficient is positive, 1.268, highly statistically significant at the 0.1% threshold), the opposite to Marino et al. [38] for BRICS countries, and the perceptions of having confidence in the rules of law (*Rule_law*) (the estimated coefficient is positive, 0.663, highly statistically significant at the 0.1% threshold). Nevertheless, the perceptions of the quality of public services and the trustworthiness into the government’s engagement in public policies (*GOV_effect*) is not statistically significant, it registered a positive result, which outlined the premises for its enforcement into the future [35].

As regards the total general government expenditures (*Gen_GOV_exp*) and the component of government contribution for environmental protection (*Env_GOV_exp*), these dimensions had favorable impacts upon the economic development of EU countries (positive estimated coefficients, although with a lower degree of statistical significance). 

Notwithstanding, jointly, implications of all considered public governance variables upon the economic development have positive spillover effects reflected by a reduction of poverty at the level of all EU countries (the estimated coefficient is negative, –0.274, extremely statistically significant at the 0.1% threshold), as Grindle [12] also substantiated.

Thus, we can say that, the fifth hypothesis, H_5_: There are overall (direct, indirect, total) significant implications of public administration credentials upon economic and social development (by reducing the poverty), is fulfilled.

### 4.3. Results of the Gaussian Graphical Models 

**Figure 5:**
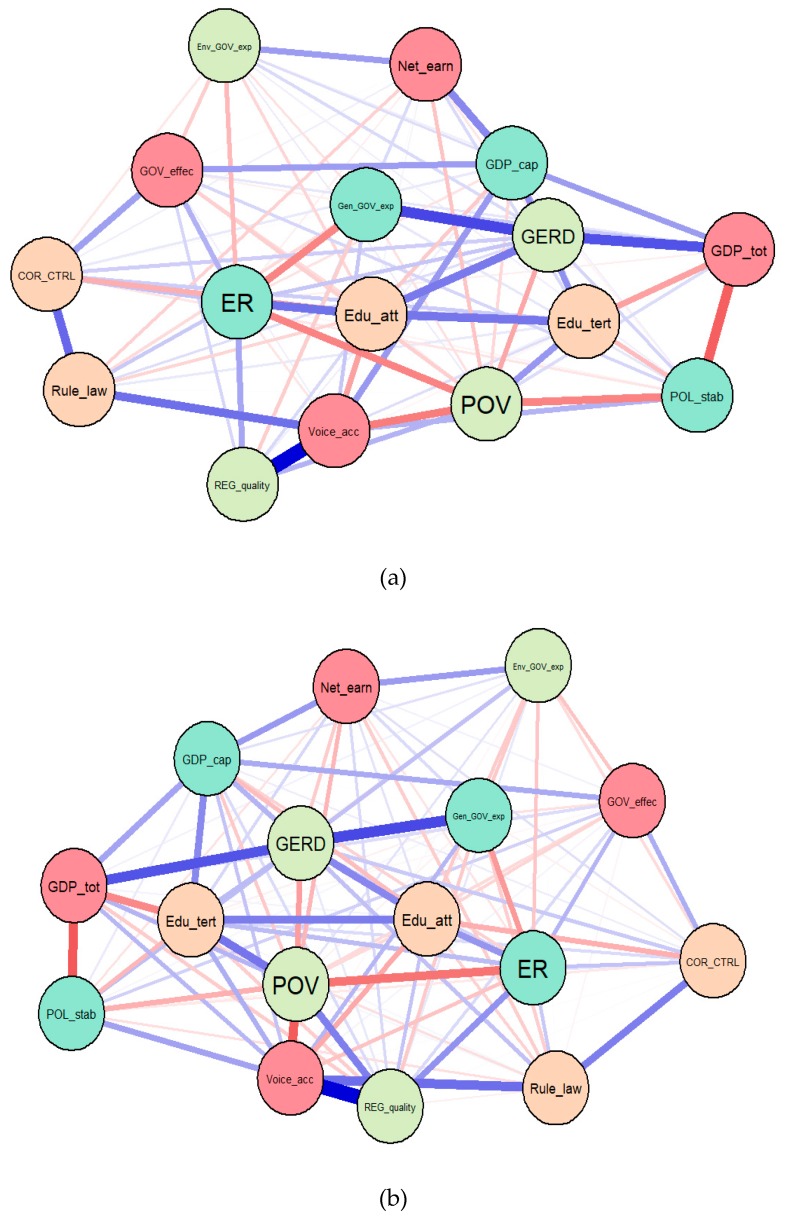
GGMs (Gaussian graphical models) for EU-28, 1995–2017: (**a**) Extended Bayesian information criterion (EBIC) and least absolute shrinkage and selection operator (LASSO), and (**b**) Partial correlation (PCOR). Source: Authors’ research in R program.

To further enhance the interlinkages between public administration dimensions and the other socio-economic variables considered in our empirical analysis within the EU, we have further deployed two Gaussian graphical, models (GGM), configured based on the extended Bayesian information criterion (EBIC) and least absolute shrinkage and selection operator (LASSO) (Figure 5a), and partial correlation (PCOR) (Figure 5b).

Both Gaussian graphical models reinforce previous SEM results and entail very strong relationships between the poverty risk *(POV)* and the perceptions of public authorities’ implications for private benefit, including different types of corruption *(COR_CTRL)*, the perceptions of the government’s ability to enforce reliable policies and regulations in order to sustain private sector development (*REG_quality*), the perceptions of people in deciding on their government, the freedom of expression, association, and media (*Voice_acc*), and the perceptions of having confidence in the rules of law (*Rule_law*). A decisive role is also played here by education (*Edu_att* and *Edu_tert*) and research and development support *(GERD)*. Another extremely relevant impact of public governance is configured upon the labor market outcomes (namely, employment rate, *ER*), which is basically enclosed by the public administration credentials, under a sheer influence of the educational attainment/background.

GDP per capita, on the other hand, is tightly connected with the government effectiveness *(GOV_effect)*, general *(Gen_GOV_exp),* and environmental *(Env_GOV_exp)* government expenditures.

Hence, the fifth hypothesis, H_5_: There are overall (direct, indirect, total) significant implications/interlinkages of public administration credentials upon economic and social development, is reinforced and fulfilled.

## 5. Conclusions

Nowadays, it has been observed that the level of quality of the public governance in EU countries has not substantially improved, despite the numerous efforts accounted by each Member State and the EU as a whole (through its fundamental bodies). On the contrary, the tendency is quite negative, which requires more attention to be given to this extremely topical subject. 

This article provides an overview of the implications of public administration on economic and social development in the EU, by analyzing the relationship between good public governance and environmental support for sustainable economic development. In terms of measurement, several macroeconomic indicators and the worldwide governance indicators (WGIs) were used to account for public governance in the EU countries during 1995–2017. Of the macroeconomic credentials and WGIs, the following indicators were considered as most relevant for the hypotheses proposed: general government expenditure share of GDP, focusing on environmental protection component, on the one hand, and the credentials of the public administration activities, namely public governance, on the other hand. As regards economic and social development, three implications were considered: enhancing GDP per capita (for economic development), reducing poverty (for the main social impact), and heightening R and D expenditures (for technological and innovation support).

The empirical analysis was based on three econometric techniques: (i) macro-econometric models—robust regression technique, in order to assess direct implications of public administration credentials (unifactorial and multifactorial models), (ii) structural equation modeling, in order to analyze the interlinkages (direct, indirect, total) between the public administration credentials and economic development (captured through welfare increases and poverty reduction), and (iii) Gaussian graphical models, designed to account for the interdependencies between all considered variables.

The main results emphasize that there are direct joint implications of each public administration dimension on economic development, research and development support, and socio-economic credentials, especially upon poverty reduction. Our results are consistent with several other studies from the literature [13,14,15,37,38].

The cumulative linkage with poverty was partially validated through the macro-econometric models (as Kwon and Kim [39] also suggested), only for the general and environmental government expenditures, government effectiveness, and political stability perceptions. SEM models further attested that the joint implications of all considered public governance variables upon the economic development have positive spillover effects, reflected by a reduction of poverty at the level of all EU countries, as Grindle [12] also substantiated. Nonetheless, bidirectional, the GDP per capita is tightly connected with government effectiveness *(GOV_effect)*, rule of law (*Rule_law*), and corruption control (*COR_CTRL*), while the utterly keen positive implications are led by the general *(Gen_GOV_exp)* and environmental *(Env_GOV_exp)* government expenditures, as attested by the results of our macro-econometric and GGM models. In terms of the government support dedicated to environmental protection and its implications on sustainable economic development of EU countries, our results are in line with Ercolano and Romano [42] (p. 1145), since the allocation of the environmental expenditures at the level of the EU strongly depend on “specific circumstances and priorities”. Thus, to enhance the impact of public environmental support on economic growth and poverty reduction, EU MS need to reconfigure actual governance policies to involve participation from multiple subjects, as Zhang and Chen [45] also suggested, “by strengthening the rule of law, improving quality of policy formulation, policy implementation, and quality of contract enforcement”, as Gil et al. [44] (p. 1111) also recommended. 

Coming to the policy implications of our research, of course general government expenditure share of GDP has a positive impact on environmental support for sustainable economic development; however, to recommend an increase of such expenditure would not represent much of a policy recommendation. There is always room for spending more money. A good policy recommendation should be cost effective. On the other hand, European States should work on the credentials of the public administration activities, namely public governance, specified as government effectiveness, rule of law, and corruption control. The first and most important step is to make it clear on the public agenda that there is a problem of public administration. Public administration is not an issue for specialists of administrative law, public administration is a crucial enabling factor of policy formulation and implementation. There is no higher priority: climate change, environmental protection, and other important items in the public agenda, they all need an effective public administration. Civil society, and especially the representatives of workers and of enterprises, should write the effectiveness of public administration on their own agenda as it appears unlikely from a logical point of view that public administration reform will be brought about only by public administration itself.

The main limitations of this research are given by the critics assigned to the WGIs, since they are based on perceptions not facts [51], as well as by the relatively low availability of data for longer time series that are essential to capture the dynamics and amplitude of public governance dimensions across the EU MS. Our future research is oriented towards assessing the bidirectional implications and interlinkages between general government spending and good public governance, focused on specific groups of countries across the EU, developed and developing ones, but also by including population dimensions, as Merloni [36] suggested. The question could be asked: What is it that generates good government effectiveness, is it really related in a perfect (Weberian) way to government expenditure? The relationship between government effectiveness and government expenditure could then be explored, separating the two sets of variables and assuming that government effectiveness and government expenditure are unrelated, thus testing an administrative behavior hypothesis (ABH)—alternative to the Weberian hypothesis—whereby bureaucracies have a level of performance of their own, depending on other factors, and independent from how much expenditure they absorb [52].

## Figures and Tables

**Table 1:** Indicators included within the econometric models.

Acronym	Details	Unit of Measure	Database
Gen_GOV_exp	Total general government expenditure	% of GDP	Eurostat
Env_GOV_exp	Expenditure of general government on environmental protection	% of GDP	Eurostat
COR_CTRL	Control of corruption grasps the “perceptions of the extent to which public power is exercised for private gain, including both petty and grand forms of corruption, as well as capture of the state by elites and private interests” [28]	Rank −2. to 2.5	The World Bank
GOV_effect	Government Effectiveness, which, “captures perceptions of the quality of public services, the quality of the civil service and the degree of its independence from political pressures, the quality of policy formulation and implementation, and the credibility of the government’s commitment to such policies” [28]	Rank −2.5 to 2.5	The World Bank
POL_stab	Political Stability and Absence of Violence/Terrorism, “measures perceptions of the likelihood of political instability and/or politically motivated violence, including terrorism” [28]	Rank −2.5 to 2.5	The World Bank
REG_quality	Regulatory Quality grasps for, “perceptions of the ability of the government to formulate and implement sound policies and regulations that permit and promote private sector development” [28]	Rank −2.5 to 2.5	The World Bank
Rule_law	Rule of Law that measure “perceptions of the extent to which agents have confidence in and abide by the rules of society, and in particular the quality of contract enforcement, property rights, the police, and the courts, as well as the likelihood of crime and violence” [28]	Rank −2.5 to 2.5	The World Bank
Voice_acc	Voice and accountability, “captures perceptions of the extent to which a country’s citizens are able to participate in selecting their government, as well as freedom of expression, freedom of association, and a free media” [28]	Rank −2.5 to 2.5	The World Bank
GDP_cap	Gross domestic product per capita	constant 2010 USD	OECD
GDP_tot	Gross domestic product total	constant 2010 USD	OECD
POV	People at risk of poverty or social exclusion	% of population	Eurostat
GERD	Research and development (R and D) expenditure	% of GDP	Eurostat
EDU_Tert	Educational attainment for tertiary education (levels 5–8)	% of the population aged 30–34	Eurostat
Edu_att	Educational attainment for upper secondary and post-secondary non-tertiary education (levels 3–4)	% of the population aged 15–64 years	Eurostat
Net_earn	Annual net earnings for, “two-earner married couple, with two children” [49]	Purchasing Power Standard (PPS)	Eurostat
ER	Employment rate, 20–64 years	% of total population	Eurostat

Source: authors’ process.

**Table 2:** Results of public administration implications upon GDP per capita within the EU, 1995–2017.

Independent Variables	(1)	(2)	(3)	(4)	(5)	(6)	(7)	(8)
	log_GDP_Cap	log_GDP_Cap	log_GDP_Cap	log_GDP_Cap	log_GDP_Cap	log_GDP_Cap	log_GDP_Cap	log_GDP_Cap
log_COR_ CTRL	0.583 *** (0.0186)							
log_GOV_ effect		0.988 *** (0.0254)						
log_POL_ stab			0.416 *** (0.0411)					
log_REG_ quality				1.170 *** (0.0446)				
log_Rule_ law					0.924 *** (0.0258)			
log_Voice_ acc						1.546 *** (0.0507)		
log_Gen_ GOV_exp							2.347 *** (0.171)	
log_Env_GOV_exp								0.144 ** (0.0552)
_cons	10.27 *** (0.0175)	10.07 *** (0.0155)	10.27 *** (0.0332)	10.02 *** (0.0226)	10.12 *** (0.0179)	10.04 *** (0.0187)	1.178 (0.648)	10.18 *** (0.0389)
*N*	479	507	511	526	498	530	634	630
*R* ^2^	0.673	0.749	0.168	0.568	0.721	0.638	0.230	0.011

Standard errors in parentheses: * *p* < 0.05, ** *p* < 0.01, *** *p* < 0.001; *N*—number of observations; *R*^2^—coefficient of determination. Source: Authors’ contribution in Stata.

**Table 3:** Results of public administration implications on poverty within the EU, 1995–2017.

Independent Variables	(1)	(2)	(3)	(4)	(5)	(6)	(7)	(8)
	log_POV	log_POV	log_POV	log_POV	log_POV	log_POV	log_POV	log_POV
log_COR_CTRL	−0.167 *** (0.0116)							
log_GOV_effect		−0.357 *** (0.0163)						
log_POL_stab			−0.302 *** (0.0175)					
log_REG_quality				−0.481 *** (0.0300)				
log_Rule_law					−0.271 *** (0.0160)			
log_Voice_acc						−0.656 *** (0.0280)		
log_Gen_GOV_exp							−0.772*** (0.101)	
log_Env_GOV_exp								0.0295 (0.0348)
_cons	3.087 *** (0.0111)	3.155 *** (0.00972)	3.030 *** (0.0144)	3.218 *** (0.0127)	3.135 *** (0.0106)	3.212 *** (0.0101)	6.074 *** (0.384)	3.150 *** (0.0203)
*N*	345	364	361	378	361	378	378	376
*R* ^2^	0.377	0.571	0.452	0.407	0.444	0.592	0.134	0.002

Standard errors in parentheses: * *p* < 0.05, ** *p* < 0.01, *** *p* < 0.001; *N*—number of observations; *R*^2^—coefficient of determination Source: Authors’ contribution in Stata.

**Table 4:** Results of public administration implications upon research and development (R and D) support within the EU, 1995–2017.

Independent Variables	(1)	(2)	(3)	(4)	(5)	(6)	(7)	(8)
	log_GERD	log_GERD	log_GERD	log_GERD	log_GERD	log_GERD	log_GERD	log_GERD
log_COR_CTRL	0.422 *** (0.0239)							
log_GOV_effect		0.680 *** (0.0344)						
log_POL_stab			0.362 *** (0.0392)					
log_REG_quality				0.766 *** (0.0493)				
log_Rule_law					0.586 *** (0.0308)			
log_Voice_acc						1.157 *** (0.0609)		
log_Gen_GOV_exp							2.679 *** (0.151)	
log_Env_GOV_exp								0.0735 (0.0521)
_cons	0.335 *** (0.0225)	0.220 *** (0.0210)	0.306 *** (0.0317)	0.111 *** (0.0250)	0.236 *** (0.0225)	0.119 *** (0.0224)	−10.02*** (0.573)	0.166 *** (0.0367)
*N*	478	506	510	525	498	529	633	629
*R* ^2^	0.395	0.437	0.144	0.316	0.422	0.406	0.333	0.003

Standard errors in parentheses: * *p* < 0.05, ** *p* < 0.01, *** *p* < 0.001; *N*—number of observations; *R*^2^—coefficient of determination. Source: Authors’ contribution in Stata.

**Table 5:** Results of public administration implications on multiple socio-economic dimensions within the EU, 1995–2017.

**Explanatory Variables**	**(1)**	**(2)**	**(3)**	**(4)**
	**log_GDP_tot**	**log_GDP_cap**	**log_POV**	**log_GERD**
log_COR_CTRL	−0.239 (0.159)	0.0501 (0.0316)	−0.0135 (0.0244)	0.0726 (0.0387)
log_GOV_effect	0.209 (0.278)	0.958 *** (0.0552)	−0.284 *** (0.0454)	0.604*** (0.0679)
log_POL_stab	−1.108 *** (0.115)	−0.0601 ** (0.0229)	−0.102 *** (0.0169)	−0.000730 (0.0281)
log_REG_quality	0.539 (0.381)	−0.0101 (0.0757)	0.0662 (0.0571)	0.207 * (0.0929)
log_Rule_law	−0.290 (0.239)	−0.0204 (0.0475)	0.0704 (0.0586)	0.0606 (0.0583)
log_Voice_acc	2.820 *** (0.567)	0.168 (0.112)	−0.280 *** (0.0774)	−0.326 * (0.138)
log_Gen_GOV_exp	4.513 *** (0.493)	0.620 *** (0.0979)	−0.342 *** (0.0697)	1.591 *** (0.120)
log_Env_GOV_exp	0.433 *** (0.122)	0.0480 * (0.0242)	−0.0843 *** (0.0195)	−0.134 *** (0.0297)
_cons	8.238 *** (1.875)	7.669 *** (0.372)	4.410 *** (0.264)	−5.862 *** (0.456)
*N*	461	461	332	460
*R* ^2^	0.391	0.811	0.613	0.690
	**(5)**	**(6)**	**(7)**	**(8)**
	**log_Edu_tert**	**log_Edu_att**	**log_ER**	**log_Net_earn**
log_COR_CTRL	0.0671 (0.0431)	0.00193 (0.0159)	0.0108 (0.00799)	0.141 ** (0.0438)
log_GOV_effect	0.286 *** (0.0751)	−0.0430 (0.0278)	0.0465 ** (0.0141)	0.598 *** (0.0752)
log_POL_stab	−0.138 *** (0.0317)	0.0604 *** (0.0116)	0.00140 (0.00587)	−0.129 *** (0.0319)
log_REG_quality	0.220 * (0.107)	0.131 *** (0.0381)	0.101 *** (0.0193)	0.242 * (0.111)
log_Rule_law	0.178 ** (0.0659)	0.0661 ** (0.0239)	0.0232 (0.0121)	−0.254 *** (0.0665)
log_Voice_acc	−0.638 *** (0.164)	−0.372 *** (0.0566)	−0.0952 *** (0.0285)	0.0864 (0.175)
log_Gen_GOV_exp	0.00803 (0.141)	0.0125 (0.0493)	−0.0540 * (0.0253)	0.336 * (0.144)
log_Env_GOV_exp	−0.106 ** (0.0342)	−0.0281 * (0.0122)	−0.0276 *** (0.00629)	0.221 *** (0.0336)
_cons	3.327 *** (0.534)	4.268 *** (0.187)	4.424 *** (0.0959)	9.129 *** (0.546)
*N*	419	457	438	414
*R* ^2^	0.295	0.164	0.441	0.530

Standard errors in parentheses, * *p* < 0.05, ** *p* < 0.01, *** *p* < 0.001; *N*—number of observations; *R*^2^—coefficient of determination. Source: Authors’ contribution in Stata.

## Appendix A

**Table A1:** Summary statistics, EU-28, 1995–2017.

Variables	*N*	Mean	SD	Min	Max
Gen_GOV_exp	634	44.75694	6.715879	26.3	65
Env_GOV_exp	634	0.724448	0.349929	−0.3	1.9
COR_CTRL	532	1.039589	0.7993224	−0.615191	2.469991
GOV_effect	532	1.140354	0.6204506	−0.5690975	2.353998
POL_stab	532	0.7999119	0.4288635	−0.4737767	1.760102
REG_quality	532	1.186079	0.4545325	−0.1844347	2.098008
Rule_law	532	1.117348	0.6240554	−0.6342322	2.100273
Voice_acc	532	1.122446	0.3433722	−0.2924617	1.800992
GDP_cap	644	29,976.13	19,970.28	3781.904	110,001.1
GDP_tot	644	5.74e+11	8.77e+11	5.56e+09	3.87e+12
POV	378	24.54021	8.072291	12.2	61.3
GERD	644	1.385272	0.8714707	0	3.914
EDU_Tert	564	30.11117	11.60818	7.4	58.7
Edu_att	640	67.18289	14.87782	17.1	88
Net_earn	590	41,122.07	36,279.75	−10,186.6	311,052
ER	585	68.57812	6.130499	51.7	81.8
*N total*	644				

Source: Authors’ research.

**Table A2:** Unit-root tests of crude variables, EU-28, 1995–2017.

Variables	Statistic			*p*-Value
	t-bar	t-tilde-bar	z-t-tilde-bar	
Gen_GOV_exp	−2.4628	−2.0926	−4.6655	0.0000
Env_GOV_exp	−2.3353	−2.0326	−4.2556	0.0000
COR_CTRL	−1.5610	−1.4237	−0.2621	0.3966
GOV_effect	−1.8705	−1.6323	−1.7033	0.0443
POL_stab	−2.7935	−2.2078	−5.6788	0.0000
REG_quality	−1.6026	−1.4433	−0.3969	0.3457
Rule_law	−1.9147	−1.6050	−1.5146	0.0649
Voice_acc	−1.9054	−1.6657	−1.9337	0.0266
GDP_cap	−1.2322	−1.1258	1.9503	0.9744
GDP_tot	−0.9110	−0.8534	3.8074	0.9998
POV	−1.9591	−1.5555	−1.6386	0.9979
GERD	−1.5676	−1.4113	0.0035	0.5014
EDU_Tert	−0.1715	−0.1494	8.5613	0.9999
Edu_att	−1.5457	−1.2821	0.8795	0.8104
Net_earn	−1.4297	−1.0637	2.3129	0.9896
ER	−0.7492	−0.7679	4.2929	0.9999

Source: Authors’ research.

**Table A3:** Cronbach’s alpha for the SEM models, EU-28, 1995–2017.

Test Scale = Mean (Standardized Items)				
Average EU-28				
Item	Obs	Sign	Interitem Correlation	Alpha
Gen_GOV_exp	634	+	0.5336	0.9115
Env_GOV_exp	630	−	0.6044	0.9322
COR_CTRL	479	+	0.4474	0.8793
GOV_effect	508	+	0.4359	0.8743
POL_stab	511	+	0.4960	0.8985
REG_quality	526	+	0.4520	0.8813
Rule_law	532	+	0.4243	0.8690
Voice_acc	530	+	0.4296	0.8714
GDP_cap	644	+	0.4509	0.8808
POV	378	−	0.4542	0.8822
Total scale			0.4725	0.8996

Source: Authors’ research.

**Table A4:** Wald tests for equations associated with the SEM models, EU-28, 1995–2017.

EU-28			
Variables	Chi2	df	*p*-Value
Log_GDP_cap	821.04	8	0.0000
Log_POV	211.39	1	0.0000

Source: Authors’ research. H0: all coefficients excluding the intercepts are 0. We can thus reject the null hypothesis for each equation.

**Table A5:** Goodness-of-fit tests for the SEM models, EU-28, 1995–2017.

EU-28		
Likelihood ratio		
chi2_ms(8)	130.724	model versus saturated
*p* > chi2	0.000	
chi2_bs(17)	708.912	baseline versus saturated
*p* > chi2	0.000	
Information criteria		
AIC	599.309	Akaike’s information criterion
BIC	649.854	Bayesian information criterion
Baseline comparison		
CFI	0.823	Comparative fit index
TLI	0.623	Tucker-Lewis index
Size of residuals		
SRMR	0.063	Standardized root mean squared residual
CD	0.711	Coefficient of determination

Source: Authors’ research.
